# Psychometric assessment of scales for measuring loneliness and social isolation: an analysis of the household, income and labour dynamics in Australia (HILDA) survey

**DOI:** 10.1186/s12955-022-01946-6

**Published:** 2022-03-05

**Authors:** Karine E. Manera, Ben J. Smith, Katherine B. Owen, Philayrath Phongsavan, Michelle H. Lim

**Affiliations:** 1 Sydney School of Public Health, Prevention Research Collaboration, Charles Perkins Centre, The University of Sydney, Research and Education Network, Western Sydney Local Health District, Westmead Hospital, Corner Darcy & Hawkesbury Roads, Westmead, NSW 2145 Australia; 2 Iverson Health Innovation Institute, Swinburne University of Technology, Melbourne, Australia

**Keywords:** Loneliness, Social isolation, Structural equation modelling, Factor analysis, Reliability

## Abstract

**Background:**

Loneliness and social isolation are increasingly recognised as global public health threats, meaning that reliable and valid measures are needed to monitor these conditions at a population level. We aimed to determine if robust and practical scales could be derived for conditions such as loneliness and social isolation using items from a national survey.

**Methods:**

We conducted psychometric analyses of ten items in two waves of the Household, Income and Labour Dynamics in Australia Survey, which included over 15,000 participants. We used the Hull method, exploratory structural equation modelling, and multidimensional item response theory analysis in a calibration sample to determine the number of factors and items within each factor. We cross-validated the factor structure using confirmatory factor analysis in a validation sample. We assessed construct validity by comparing the resulting sub-scales with measures for psychological distress and mental well-being.

**Results:**

Calibration and cross-validation consistently revealed a three-factor model, with sub-scales reflecting constructs of loneliness and social isolation. Sub-scales showed high reliability and measurement invariance across waves, gender, and age. Construct validity was supported by significant correlations between the sub-scales and measures of psychological distress and mental health. Individuals who met threshold criteria for loneliness and social isolation had consistently greater odds of being psychologically distressed and having poor mental health than those who did not.

**Conclusions:**

These derived scales provide robust and practical measures of loneliness and social isolation for population-based research.

**Supplementary Information:**

The online version contains supplementary material available at 10.1186/s12955-022-01946-6.

## Introduction

Loneliness and social isolation are increasingly recognised as global public health threats. Loneliness is described as the negative subjective appraisal of social relationships, elicited by feeling like one’s social relationships fail to adequately meet one’s social needs [1]. Social isolation has been described as the absence of social interactions, often measured in terms of the size and frequency of contacts [2]. Studies have shown that loneliness and social isolation are significant contributors to higher rates of premature mortality, with increased risks of 26% and 29%, respectively, compared with people who are not lonely or isolated [3]. In addition to the adverse effects of loneliness and social isolation on physical health and mortality, studies have also shown strong associations between loneliness, social isolation and poor mental health outcomes [4]. These relationships are such that measures of mental health and mental well-being have been used to test the construct validity of loneliness and social isolation scales [5, 6].

Given the growing body of evidence that loneliness and social isolation are important but neglected public health risk factors [7], there is a strong need to develop psychometrically validated and sound measures that can be used to identify and monitor the prevalence, distribution and trends in these conditions at the population level. Using reliable and valid instruments is therefore paramount for understanding the extent of loneliness and social isolation, and their subsequent impact on health and health-related behaviours. However, measuring the prevalence and distribution of these conditions in populations has been historically difficult. Population-based surveys, conducted as either telephone interviews or self-complete questionnaires, commonly involve the collection of a whole sweep of measures and can be lengthy and laborious to complete. Respondents are vulnerable to survey fatigue resulting in inaccuracies in responses to questions, hence brief measures are often favoured to ensure that respondents remain engaged [8]. For the assessment of constructs such as loneliness or social isolation, the use of longer scales (i.e., greater than 10 items) has been recommended for individual-level assessment in clinical settings, while shorter scales are found to be particularly beneficial for providing broad, population-level estimates, and for guiding public health and policy responses [9].

Although loneliness and social isolation are widely used constructs, different understandings about their nature and attributes are reflected in the variety of short- and long-form measures of these conditions being used in clinical and population surveys [10, 11]. Commonly used measures of loneliness include the 20-item Revised UCLA Loneliness Scale [12], the 11-item De Jong Gierveld loneliness scale [13] and in social isolation, the 10-item Lubben Social Network Scale [14], and Berkman Social Network Index [15]. Shortened versions of these scales have been developed for use in large surveys, however these have all been developed and tested in surveys of older adults and infrequently used in surveys that are representative of the general population [16–18].

The diversity of scales and samples when measuring loneliness and social isolation has led to varying findings in the literature. For example, loneliness is shown to affect all age groups, but the prevalence has been reported as highest among both young adults (18–29) and older adults (65–79) [19, 20]. Other studies have shown that older adults (65–89) are less lonely compared to other age groups [21], but these contrasting findings are likely due to limitations in sample size and population samples. Broadly speaking, it is recognised that the social groups at greater risk of both loneliness and social isolation include ethnic minorities, lesbian, gay, bisexual and trans + (LGBTIQ +) people, people with disabilities or chronic health conditions, caregivers, and non-community-dwelling older people [22, 23].

While many of the existing scales have been psychometrically validated, the heterogeneity of scales and items within them suggests that they may not be based on theoretical frameworks of loneliness and social isolation. As constructs, both loneliness and social isolation are recognised as social deficits which sit on the low end of what’s been termed the social connection continuum [24]. Key theoretical distinctions have been made between the two, with loneliness being characterised as a feeling of distress or social pain which accompanies the perception that social needs are not being met [25, 26]. Social isolation, on the other hand, focuses more on the quantity and quality of social interactions, incorporating how one interacts with their social environment and networks [26]. Developing or establishing measures for these constructs requires both empirical and data-driven evidence as well as a theoretical basis which aligns with the current conceptualisation of these issues. The Household Income and Labour Dynamics in Australia (HILDA) survey is a nationally representative panel study of over 9,000 Australian households conducted annually, with wave 1 of the survey starting in 2001 [27]. The HILDA survey follows participants from age 15 years onwards and collects data on household and family relationships, income, employment, health, and education. While the HILDA self-completion questionnaire includes a number of items concerning social interactions and social support, previous estimates of the prevalence of loneliness among study participants have been derived from responses to the single-item statement “*I often feel very lonely*” [28]. This is consistent with several other large surveys that have used single-item measures for loneliness [29, 30]. While efficient, these single-item measures may be subjected to response bias, as loneliness is well-known to be associated with stigma, social desirability bias and gender bias (i.e., men are less likely to report loneliness than women [31–33]. The finding that individuals respond differently to one-item direct measures such as *Are you lonely?* as opposed to *Would you like company* [34]*?* also highlights the challenges in measuring this construct.

In light of these complexities, it is optimal to measure loneliness and other dimensions of social support and relationships using multiple items as opposed to a single item [35]. We aimed to undertake psychometric analysis of items examining social support in the HILDA survey, to determine if robust and practical scales could be derived for conditions such as loneliness and social isolation within this large population study.

## Methods

### Participants

Data used in this study were collected from the HILDA survey. In this cohort study a number of household-level and person-level questionnaires are completed for each household. The present study uses data from the self-completion questionnaire (herein called ‘HILDA questionnaire’), which collects person-level data and is undertaken by individuals aged 15 years or older in each participating household. Approximately 15,000 individuals respond to the HILDA questionnaire each year. In this study we included individuals who completed the questionnaire in waves 17 (2017) and 19 (2019), which were chosen to provide a contemporary sample and to assess the consistency of psychometric properties across time.

### Measures

The HILDA self-completion questionnaire collects comprehensive information about a person’s general health and well-being, lifestyle and living situation, finances, work, and parenting. This instrument includes 10 items in which participants are required to describe and appraise their social support, by responding on a 7-point Likert scale from 1 (strongly disagree) to 7 (strongly agree) to a sequence of statements. Seven of the 10 items come from Henderson, Duncan-Jones [36] and three from Marshall and Barnett [37]. In previous analyses of the HILDA survey data these items have been used as a single scale for measuring social support and social networks [38, 39], but to our knowledge this scale has not been validated. A list of the 10 items is included in Additional file 1: Fig. S1.

Each wave of the HILDA questionnaire includes measures of mental well-being and psychological distress which we utilised for the purpose of assessing construct validity. Psychometric studies of loneliness scales have shown that positive and negative affect (including mental well-being and distress) are related constructs with moderate correlations to loneliness and are suitable for validation purposes [40]. In this study, we used the 36-item Short Form Survey (SF-36) Mental Component Summary (MCS) as a measure of mental health status [41]. The full SF-36 survey instrument and guide to computing the MCS are freely available online. In brief, the MCS is calculated in a three-step process involving: (1) standardising each of the eight SF-36 health domains using a Z-score transformation of means and standard deviations from the 1995 Australian National Health Survey (NHS) [42]; (2) aggregating Z-scores using coefficients from the NHS as weights; and (3) transforming scores to have a mean of 50 and a standard deviation of 10. Individuals with MCS scores of 42 or below, which has proven to be a cut-point indicative of clinical depression, are classified as having poor mental health [43].

The other measure used for validation purposes was the Kessler Psychological Distress Scale (K10), which has been included in every second wave of the HILDA survey from 2007 [44]. The K10 provides a measure of non-specific psychological distress based on questions about negative emotional states experienced in the past 4-week period. Scores on the K10 range from 10 to 50 with higher scores indicating higher levels of psychological distress. Designated cut-off scores have previously been assigned as low to moderate (10–21) and high to very high (22–50) levels of psychological distress. In this study, we assigned those scoring 22 or higher on the K10 as having psychological distress [44–46].

To include a contemporary sample of participants who have data for the 10 social support items, SF-36 and K10, we included all individuals who completed the self-completion questionnaire in waves 17 and 19.

### Statistical analysis

We calculated descriptive statistics, including frequencies and proportions, for individuals across the two waves. For each of the two waves we randomly allocated participants into a calibration sample (wave 17 n = 7869; wave 19 n = 7549) and validation sample (wave 17 n = 7549; wave 19 n = 7705). Using the calibration samples, we identified the number of factors and items within each factor based on results from the Hull method, exploratory structural equation modelling, and multidimensional item response theory analysis. Negatively worded items (items 1, 2, 4, 5 and 7) were reverse coded for all analyses so that a high value indicates the same type of response on every item. We evaluated the resulting factor structure using confirmatory factor analysis on the validation samples. Analyses were conducted using R software version 4.0.4, SPSS version 25 and flexMIRT version 3.0.

#### Factor structure and item selection

The Hull method identifies a model with an optimal balance of model fit and number of parameters [47]. This method (HULL function in package ‘EFAtools’) was used to determine the number of factors to retain, with principal axis factoring (PAF) set as the extraction method due to the skewness of the data [48] and eigen types based on exploratory factor analysis (EFA).

Exploratory structural equation modelling (ESEM) can be used to investigate the structure of factors, and does so by integrating features of EFA and confirmatory factor analysis (CFA) [49]. ESEM builds on these techniques by permitting the specification of the expected factor number, which in this study is based on findings from the Hull method and theoretical consideration, while allowing all cross-loadings (i.e. items can have non-zero loadings across factors, which is inherent in psychological measurement) [50]. ESEM was run with robust maximum likelihood and geomin rotation using the ‘esem’ function in package ‘lavaan’. Items with factor loadings < 0.40 were omitted if there was no theoretical justification for retaining them.

Item response theory is a framework for model-based measurement of items, whereby responses are modelled as a function of individual respondent characteristics and item properties [51]. While traditional item response theory is limited by the assumption of unidimensionality (i.e. that there is one dominant latent trait being measured and that this drives the responses observed for each item in the measure), multidimensional item response theory (MIRT) extends both unidimensional item response theory and factor analysis, enabling the analysis of multiple constructs simultaneously [52]. We ran a MIRT graded response model using flexMIRT software [53] which is used for polytomous responses such as Likert scales [54], to examine the extent to which the items differentiate among individuals across different degrees of the underlying traits. The slope parameter (*a*) refers to the item’s discriminative ability, with higher values indicating a stronger association with the construct [55], and the intercept (*d*) refers to the log-odds of responding above a given category when the latest trait θ = 0. Items with a slope parameter (*a*) between 1.35 and 1.70 were considered to have high discrimination and above 1.70 considered to have very high discrimination [56]. The information from these analyses and theory were combined to select a final pool of items for each of the factors.

#### Validation of the factor structure

Once the number of factors and item selection were established using the calibration samples of waves 17 and 19, we cross-validated the factor structure using CFA in the validation samples of both waves. CFA tests whether the data fit the hypothesised factor structure by constraining the correlations between factors to zero. Model fit measures are then obtained to assess how well the proposed model captures the covariance between all the items in the model, with comparative fit index (CFI) > 0.90, root mean square error of approximation (RMSEA) < 0.08 and standardized root mean square residual (SRMR) < 0.08 indicative of good fit [57]. The CFA was run using the ‘lavaan’ package with maximum likelihood robust (MLR) to address non-normality.

#### Measurement invariance of the adapted scales

We evaluated measurement invariance of the scale across time (waves 17 and 19), gender and age groups. In a stepwise process, we tested configural (i.e., whether the factor structure was the same across groups), metric (i.e., whether factor loadings were the same across groups), scalar (whether the mean structures/intercepts of items were the same across groups) and strict invariance (i.e., whether the residual error was the same across groups). Configural invariance was established with a CFI > 0.90 and RMSEA < 0.06. Metric, scalar and strict invariance were established when ΔCFI was < 0.01 and when Δ χ^2^ was non-significant (*p* > 0.05) [58]. Partial metric and/or scalar measurement invariance was established when at least two factor loadings and/or intercepts per latent factor were invariant.

#### Internal consistency of the adapted scales

We tested the internal consistency of the identified scales by generating Cronbach’s alpha (α) and McDonald’s omega (ω) using the ‘psych’ package. While Cronbach’s α is the more widely used measure of internal consistency, it is based on the assumption of tau-equivalence i.e., that all items have equal factor loadings. McDonald’s ω, instead, has the advantage of accounting for the strength of association between items by assuming a congeneric model, i.e., factor loadings may differ between items. This corrects for under- and overestimations from Cronbach’s α [59]. Recommended cut-offs for α and ω are the same, with a coefficient of 0.70 considered the minimal level for a usable scale for group-based measurements [60].*Construct Validity: The Association of the Adapted Scales with Psychological distress and mental well-being*

To assess construct validity, we examined non-parametric correlations between the adapted scales with the K10 and MCS, respectively, using Spearman’s rank order correlations. To assess the performance of the scales in discriminating differences in psychological distress and poor mental health, using the K10 threshold of 22 or higher and MCS score of 42 or less, respectively, we then examined the area under the curve (AUC) generated by non-parametric Receiver Operating Characteristic (ROC) analysis in SPSS. Based on examination of item scores within scales and theoretical justification, we developed a cut-off score to classify individuals within each scale as expressing that trait or not. We conducted univariable logistic regression to identify the odds of an individual having psychological distress or poor mental health if they expressed the trait.

## Results

### Sample characteristics

There were 15,637 respondents with complete data for the 10 social support items included in the HILDA questionnaire in wave 17 and 15,693 in wave 19. As shown in Table 1, sample characteristics were similar across each wave. The average age of respondents was 46 years, 47% were male, 10% spoke a language other than English and 30% had a long-term health condition.

**Table 1:** Characteristics of HILDA respondents in waves 17 and 19

Item	Wave 17 (N = 15,637)	Wave 19 (N = 15,693)
Age, *M* (SD)	45.5 (18.9)	46.1 (19.1)
Male, *n* (%)	7355 (47.0)	7370 (47.0)
Speak language other than English, *n* (%)^a^	1569 (10.0)	1544 (9.8)
Long term health condition, *n* (%)^a^	4670 (29.9)	4586 (29.2)
*Marital status, n (%)*^a^		
Married/de facto	10,055 (64.3)	9398 (59.9)
Never married	3577 (22.9)	3589 (22.9)
Divorced/separated	1353 (8.7)	1984 (12.6)
Widowed	651 (4.2)	722 (4.6)
*Employment status, n (%)*^a^		
Full time	6649 (42.5)	6702 (42.8)
Part time	3267 (20.9)	3246 (20.7)
Retired	3061 (19.6)	3213 (20.5)
Unemployed/other	2639 (16.9)	2513 (16.0)
*Household structure, n (%)*		
Lone person	2421 (15.5)	2477 (15.8)
Lone person with child	433 (2.8)	426 (2.7)
Couple with child	3642 (23.3)	3607 (23.0)
Couple without child	4985 (31.9)	5078 (32.4)
Other	4156 (26.6)	4105 (26.2)
*SEIFA quintile, n (%)*^a^		
1–2 (most disadvantaged)	2928 (18.7)	2907 (18.5)
3–4	3105 (19.9)	3072 (19.6)
5–6	3058 (19.6)	3132 (20.0)
7–8	3270 (20.9)	3309 (21.1)
9–10 (least disadvantaged)	3271 (20.9)	3261 (20.8)
SF-36 MCS score, *M* (SD)^a^	48.0 (10.9)	47.3 (11.3)
SF-36 MCS score ≤ 42, *n* (%)^a^	3673 (24.2)	4043 (26.3)
Psychological distress (K10) score, *M* (SD)^a^	16.4 (6.9)	16.8 (7.2)
Psychological distress (K10) score ≥ 22, *n* (%)^a^	2895 (18.5)	3151 (20.1)

SEIFA, Socio-Economic Indexes for Areas; MCS, Mental Component Summary; K10 score ≥ 22 corresponds to psychological distress; MCS ≤ 42 corresponds to poor mental health

^a^Missing data

### Factor structure and item selection

The Hull method indicated that a model with three factors provided the best fit in both waves 17 and 19 (Additional file 1: Figs. 2a and 2b). Therefore, we fitted a three-factor model using ESEM and a MIRT model with three dimensions to select items. Factor loadings and model fit statistics from the ESEM in waves 17 and 19 are provided in Table 2, as are results from the MIRT graded response model with three dimensions in wave 19. The MIRT model indicated that all items had high discrimination on their respective factor in both waves.

**Table 2:** Item loadings and fit statistics from ESEM and MIRT models with three factors

	Wave 17			Wave 19												
	ESEM			ESEM			MIRT									
Item	F1 (λ)	F2 (λ)	F3 (λ)	F1 (λ)	F2 (λ)	F3 (λ)	*a*1	*a*2	*a*3	*d1*	*d2*	*d3*	*d4*	*d5*	*d6*	*d7*
People don’t come to visit me as often as I would like	1.15	− 0.06	0.04	1.16	− 0.07	0.02	1.28	0.00	0.00	11.63	3.86	2.5	1.26	0.08	− 0.76	− 2.15
I often need help from other people but can’t get it	0.86	0.14	0.32	0.89	0.13	0.30	1.64	0.00	0.00	13.19	5.43	4.39	3.39	2.35	1.41	− 0.51
I don’t have anyone that I can confide in	0.38	0.17	1.01	0.17	0.06	1.30	0.00	2.24	0.00	19.64	7.83	6.11	4.95	3.64	2.38	− 0.22
I have no one to lean on in times of trouble	0.03	0.01	1.61	0.08	0.03	1.38	0.00	3.75	0.00	31.35	12.3	10.19	8.45	6.45	4.47	0.33
There is someone who can always cheer me up when I’m down	0.36	**0.77**	0.18	0.29	0.82	0.24	0.00	0.00	1.40	12.29	4.57	3.61	2.78	1.79	0.73	− 1.24
I often feel very lonely	0.98	0.19	0.29	0.85	0.19	0.43	1.40	0.00	0.00	12.41	4.64	3.4	2.48	1.48	0.75	− 0.84
I enjoy the time I spend with the people who are important to me	− 0.01	0.76	0.07	0.01	0.78	0.01	0.00	0.00	1.52	13.18	6.56	5.96	5.26	4.16	2.84	0.37
When something’s on my mind, just talking with the people I know can make me feel better	− 0.02	1.15	0.04	− 0.01	1.15	0.07	0.00	0.00	1.98	15.18	7.08	5.68	4.58	2.93	1.31	− 1.33
When I need someone to help me out, I can usually find someone	0.46	0.92	0.19	0.46	0.90	0.22	0.00	0.00	2.65	18.37	7.36	5.99	4.77	3.15	1.45	− 1.53
Chi-square	234.63			243.63			–									
CFI	0.992			0.992												
TLI	0.977			0.975												
RMSEA (90% CIs)	0.049 (0.043, 0.054)			0.051 (0.045, 0.056)			–									
SRMR	0.013			0.012												

F1–F3, factors 1 to 3; λ, standardized factor loading; a, slope; d, intercept; CFI, Comparative Fit Index; RMSEA, Root Mean Square Error of Approximation; SRMR, Standardized Root Mean Square Residual; TLI, Tucker Lewis index

Factor one included three items (item 1: “*People don’t come to visit me as often as I would like*”, item 2: “*I often need help from other people but can’t get it*” and item 7: “*I often feel very lonely*”), reflecting the concept of loneliness in terms of expressing a feeling or perception of social needs not being met.

The second factor included four items (item 6: “*There is someone who can always cheer me up when I’m down*”, item 8: “*I enjoy the time I spend with the people who are important to me*”, item 9: “*When something’s on my mind, just talking with the people I know can make me feel better*” and item 10: “*When I need someone to help me out, I can usually find someone*”).Considering these items in light of existing constructs, we found that this grouping reflected the concept of social isolation, such that they incorporate how one engages with and accesses their social environment. The items included in the loneliness and social isolation sub-scales are summarized in Additional file 1: Table S1.

Factor three included items 4: “*I don’t have anyone that I can confide in*” and 5: “*I have no one to lean on in times of trouble*”. The use of two items to identify an underlying construct has been recognized as problematic, with true-score theory indicating that more items lead to better construct validity and reliability [61, 62]. Analysis of this two-item sub-scale was therefore discontinued.

Item 3: “*I seem to have lots of friends*” was omitted from further analyses due to low loadings (0.38) and theoretically not reflecting the concept of loneliness or social support (i.e. expressing neither an appraisal of one’s feelings nor level of engagement).

### Validation of factor structure

CFA results indicated good fit of the three-factor model, with a CFI of 0.953 in wave 17 and 0.955 in wave 19, and a SRMR of 0.050 in wave 17 and 0.048 in wave 19. Model fit statistics for the CFA are also provided in Table 3.

**Table 3:** Fit statistics from the CFA model with three factors

	Wave 17	Wave 19
Chi-square	1320.78	1267.92
CFI	0.953	0.955
TLI	0.929	0.932
RMSEA (CIs)	0.083 (0.080, 0.087)	0.082 (0.078, 0.086)
SRMR	0.050	0.048

### Measurement invariance

Measurement invariance for the three-factor scale across waves, gender and age are shown in Table 4. Results showed evidence for full configural, metric, scalar and strict invariance across waves 17 and 19. In Wave 19, we found evidence for configural and partial scalar invariance across gender. There was also evidence for configural and partial metric invariance across age.

**Table 4:** Measurement invariance for the three-factor model across waves and gender

	AIC	aBIC	χ^2^	Δ χ^2^	*p*-value	CFI	ΔCFI	RMSEA	ΔRMSEA	SRMR	ΔSRMR
*Across waves (17 and 19)*											
Configural invariance	933,079.20	933,399.13	4931.2			0.95		0.07		0.04	
Metric invariance	933,074.19	933,363.16	4938.2	7.0	0.60	0.95	0.00	0.06	-0.01	0.04	0.00
Scalar invariance	933,068.25	933,326.27	4944.2	6.0	0.42	0.95	0.00	0.06	0.00	0.04	0.00
Strict invariance	933,069.55	933,281.12	4963.5	19.3	0.58	0.95	0.00	0.06	0.00	0.04	0.00
*Across sex in Wave 19 (male and female)*											
Configural invariance	427,284.77	427,556.50	2353.1			0.95		0.07		0.04	
Metric invariance	427,332.53	427,577.96	2412.9	59.8	< 0.001	0.94	− 0.01	0.07	0.00	0.05	0.01
Partial metric invariance	427,383.94	427,642.52	2458.3	45.4	< 0.001	0.94	0.00	0.07	0.00	0.05	0.00
Scalar invariance	427,597.94	427,817.08	2690.3	232.0	< 0.001	0.94	0.00	0.07	0.00	0.05	0.00
Partial scalar invariance	427,343.91	427,576.19	2413.3	0.4	0.79	0.94	0.00	0.07	0.00	0.05	0.00
Strict invariance	427,769.40	427,949.09	2879.8	466.5	< 0.001	0.94	0.00	0.06	0.01	0.07	0.02
*Across age categories*											
Configural invariance	459,166.47	459,834.67	2858.5			0.95		0.07		0.05	
Metric invariance	459,288.70	459,849.99	3028.7	170.2	< 0.001	0.95	0.00	0.07	0.00	0.05	0.00
Partial metric invariance	459,170.39	459,802.96	2869.0	10.5	0.08	0.94	− 0.01	0.07	0.00	0.05	0.00
Scalar invariance	459,616.90	460,071.27	3404.9	535.9	< 0.001	0.94	0.00	0.07	0.00	0.06	0.01
Partial scalar invariance	460,232.25	460,472.81	2602.2	− 266.8	< 0.001	0.94	0.00	0.07	0.00	0.06	0.00
Strict invariance	460,800.67	460,983.31	3196.6	594.4	< 0.001	0.94	0.00	0.07	0.00	0.06	0.00

AIC, Akaike information criterion; aBIC, sample size corrected Bayesian information criterion; CFI, comparative fit index; RMSEA, root mean square error of approximation; SRMR, standardised root mean squared residual; Δ, change

### Internal consistency

The Cronbach’s α and McDonald’s ω of the loneliness and social isolation scales in each of waves 17 and 19 are shown in Table 5. Across the two waves, both scales demonstrated good reliability, with the α and ω coefficients of 0.70 and 0.71 for the 3-item loneliness scale, respectively, in both wave 17 and wave 19. The 4-item social isolation scale reported respective α and ω coefficients of 0.80 and 0.81, respectively for both wave 17 and wave 19.

**Table 5:** Internal consistency of the loneliness and social isolation scales

	Loneliness 3-item		Social Isolation 4-item	
	Wave 17	Wave 19	Wave 17	Wave 19
Cronbach’s α	0.70	0.70	0.80	0.80
McDonald’s ω	0.71	0.71	0.81	0.81

### Construct validity

The loneliness and social isolation scales were significantly positively correlated with each other at Wave 17 and Wave 19 (0.44 and 0.43, respectively). The loneliness and social isolation scales were also correlated significantly with the K10 and MCS. We saw a positive correlation with the loneliness sub-scale scale and K10, with correlation coefficients of 0.48 across the two waves. We found a negative correlation between the loneliness sub-scale and the MCS, with a consistent coefficient of − 0.46. In contrast, the positively worded social isolation scale correlated negatively with the K10 and positively with the MCS, with coefficients of − 0.37 and 0.38, respectively (Table 6).

**Table 6:** Spearman’s correlations, area under the curve and odds ratios for loneliness and social isolation scales

	Wave 17		Wave 19	
	K10	MCS	K10	MCS
*Spearman’s correlations*^a^				
Loneliness	0.48	− 0.46	0.48	− 0.46
Social isolation	− 0.37	0.38	− 0.38	0.38
*Receiver operating characteristic: area under the curve (AUC)*^b^				
Loneliness	0.78	0.75	0.77	0.74
Social isolation	0.73	0.70	0.73	0.70
*Odds ratios—logistic regression (95% CIs)*^a,b^				
Loneliness (cut-off > 4)	6.14 (5.58–6.75)	5.57 (5.07–6.12)	5.60 (5.10–6.14)	5.07 (4.62–5.55)
Social isolation (cut-off < 4)	4.67 (4.09–5.33)	3.99 (3.49–4.56)	4.74 (4.15–5.42)	4.24 (3.70–4.85)

^a^All significant at the 0.01 level

^b^Using K10 threshold for psychological distress ≥ 22 and MCS poor mental health ≤ 42

The AUC for the loneliness scale showed fair performance, with areas ranging from 0.77 to 0.78 against the K10 psychological distress scores, and from 0.74 to 0.75 for the MCS. Similarly, the AUC for the social isolation scale was consistently fair against the K10 and MCS, with coefficients of 0.73 and 0.70, respectively (Table 6).

The threshold for classification of loneliness was determined to be a median item score of less than 4, and for social isolation a median item score of greater than 4, as this represents having a majority level of agreement (for loneliness items) or disagreement (for social isolation items).

The results of the univariable logistic regression to assess the relationship between dichotomous loneliness and social isolation variables and the categorical indicators of psychological distress and poor mental health (from the K10 and MCS), are shown in Table 6. Using the threshold median score for loneliness of less than 4, we found that 15% of respondents were lonely in waves 17 and 19. For social isolation, a threshold median score of greater than 4 resulted in 6% of respondents being classified as socially isolated in each of the waves. Across the two waves, we found that individuals classified as lonely were approximately six times more likely to be psychologically distressed than non-lonely participants, and 5 times more likely to have poor mental health. Similarly, those classified as socially isolated were approximately 4.5 times more likely to be psychologically distressed than those not socially isolated, and four times more likely to have poor mental health.

## Discussion

In this report, we identified measures for loneliness and social isolation from 10 items used to measure social support in a large, population-based cohort study. The loneliness and social isolation scales demonstrated good measurement invariance, reliability and construct validity when compared with measures for psychological distress and mental health.

We found that the first factor reflecting loneliness contained negatively worded items, which is a pattern previously observed in psychometric analyses of loneliness scales [63]. However, the three items in the loneliness scale are consistent with previous theory and literature on loneliness, which recognises it as the *subjective* evaluation of the current state of their relationships. Social isolation, related to the amount of contact a person has with others, is reflected in the four-item scale of the second factor which assesses the availability and utilisation of social relationships [64]. Although the social isolation sub-scale items were found to be all positively worded, not all the positive items possessed sufficient loading to be included in the second component (i.e., item 3 was omitted due to low loadings). While it is possible that these scales were subject to acquiescence bias, the participants in the HILDA survey completed the 10 items together (mix of positively and negatively worded items), which is a known method for controlling the bias [65]. Further, not all the negatively worded items loaded together, with items 4 and 5 loading onto their own factor separate to the other negatively worded items.

Investigation of measurement invariance found agreement of the scales across waves. Between males and females, partial scalar invariance was found. Females had higher intercepts (means) for item 9: “*When something’s on my mind, just talking with the people I know can make me feel better*”, whereas males had higher intercepts for item 7: “*I often feel very lonely*” and item 10: “*When I need someone to help me out, I can usually find someone*”. For comparisons where full measurement invariance was established (i.e., across waves), researchers can compare the observed means. However, for comparisons where full measurement invariance was not established (i.e., across gender and age), researchers should use the latent structure, latent means, correlations and prediction relations with other variables in the HILDA dataset [66].

Inclusion of the K10 psychological distress scale and MCS derived from the SF-36 in the HILDA survey enabled comparisons between these and the loneliness and social isolation scales. We found that loneliness and social isolation were consistently and significantly correlated with psychological distress and poor mental health across the waves, and that loneliness was a slightly better predictor of these outcomes than social isolation. Recent studies have similarly shown that loneliness is associated with a greater risk of mental health problems and severe depression compared to other similar constructs such as social support [4, 67, 68]. This consistency with other studies further supports the validity of the scales as measures of their respective constructs.

Given the significant impact of loneliness and social isolation on mental and physical health, population level monitoring is needed to be able to assess trends in these important social conditions, identify priority groups, and monitor the impact of policies and programs [7]. To achieve this, the scales need to be practical and ideally be able to generate prevalence estimates for those at greatest risk. The Government of the United Kingdom has played a leading role in loneliness research, policy and monitoring, internationally, and has recognised that inconsistent measures reduce the ability to compare data and limits our understanding of these critical issues [69]. Consistent measures for loneliness and social isolation are needed across different research areas, including population studies, in order to identify and monitor these critical social, community and public health problems [70].

The ongoing COVID-19 pandemic has also placed a spotlight on the need for population-level monitoring of loneliness and isolation, as social interactions have been restricted due to regulations around physical and social distancing, self-isolation and quarantining [71]. Recent evidence from the UK and USA suggests the prevalence of isolation and loneliness was high during COVID-19, particularly in the acute phase of the outbreak [72, 73]. However, the different measures used, lack of accurate pre-COVID loneliness data and lack of data from other affected countries has hindered our ability to assess the impact of COVID-19 on loneliness and isolation.

This study has a number of strengths, including the robust examination of the psychometric properties of items completed by over 15,000 participants in two waves of the HILDA study. The large sample size enabled us to determine the number of factors and items using recommended analytical techniques including the Hull method, which has shown to outperform traditional methods of factor analysis [47]. We then demonstrated the robustness of our findings through cross-validation in another sample using CFA and by testing longitudinal and cross-sectional measurement invariance. Inclusion of widely used measures of mental health and psychological distress in the HILDA survey permitted rigorous validation of the loneliness and social isolation measures. However, this study has some limitations. First, the survey was conducted in English only, with participants tending to have higher socioeconomic status. We were unable to test the sensitivity and responsiveness of the measures, so their suitability as intervention evaluation measures is not known. While this study included a large sample from a nationally representative survey, further validation will be required for use in other countries.

## Conclusions

The growing attention to loneliness and social isolation worldwide, and their known associations with morbidity and mortality [3, 74], calls for improved tools to measure the prevalence and burden of these conditions. The present study provides an important contribution by identifying and assessing the psychometric properties of two short self-report scales for loneliness and social isolation in Australia. Our findings demonstrate that these scales provide valid and reliable measures for loneliness and social isolation that are suitable for use in population surveys to improve future research and population monitoring.

### Supplementary Information

**Additional file 1: Table S1**. Items included in the loneliness and social isolation sub-scales. **Figure 1**. Ten items used to measure social interactions and support in the HILDA survey. **Figure 2a**. Results of the Hull Method in wave 17 (calibration sample). **Figure 2b**. Results of the Hull Method in wave 19 (calibration sample)

## Data Availability

The dataset supporting the conclusions of this article are available to researchers living in Australia or overseas through the National Centre for Longitudinal Data Dataverse. Information about applying for access to the data is available at: https://dataverse.ada.edu.au/dataverse/ncld.
