# Correction: Psychometric assessment of scales for measuring loneliness and social isolation: an analysis of the household, income and labour dynamics in Australia (HILDA) survey

**DOI:** 10.1186/s12955-023-02193-z

**Published:** 2023-09-29

**Authors:** Karine E. Manera, Ben J. Smith, Katherine B. Owen, Philayrath Phongsavan, Michelle H. Lim

**Affiliations:** 1 Sydney School of Public Health, Prevention Research Collaboration, Charles Perkins Centre, The University of Sydney, Research and Education Network, Western Sydney Local Health District, Sydney School of Public Health, Prevention Research Collaboration, The University of Sydney, Westmead Hospital, Corner Darcy & Hawkesbury Roads, 2145 Westmead, NSW Australia; 2 Iverson Health Innovation Institute, Swinburne University of Technology, Melbourne, Australia

**Health and Quality of Life Outcomes (2022) 20:40**

10.1186/s12955-022-01946-6

The original article [1] contains two errors in the following two sentences in the Results section, specifically for the phrases ‘greater than 4’:

‘The threshold for classification of loneliness was determined to be a median item score of less than 4, and for social isolation a median item score of greater than 4, as this represents having a majority level of agreement (for loneliness items) or disagreement (for social isolation items).’

‘For social isolation, a threshold median score of greater than 4 resulted in 6% of respondents being classified as socially isolated in each of the waves.’

The two phrases should instead state ‘less than 4’ as demonstrated in the two following amended sentences:

‘The threshold for classification of loneliness was determined to be a median item score of less than 4, and for social isolation a median item score of less than 4, as this represents having a majority level of agreement (for loneliness items) or disagreement (for social isolation items).’

‘For social isolation, a threshold median score of less than 4 resulted in 6% of respondents being classified as socially isolated in each of the waves.’

