# Supplementary material for: Psychometric assessment of scales for measuring loneliness and social isolation: an analysis of the household, income and labour dynamics in Australia (HILDA) survey
Source: Health Qual Life Outcomes. 2022 Mar 5;20:40. doi: 10.1186/s12955-022-01946-6 (PMC8897757; doi:10.1186/s12955-022-01946-6)

**Supplementary Material**

**Supplementary Table 1.** Items included in the loneliness and social isolation sub-scales

| **Loneliness** |
| --- |
| People don’t come to visit me as often as I would like |
| I often need help from other people but can’t get it |
| I often feel very lonely |
| **Social isolation** |
| There is someone who can always cheer me up when I’m down |
| I enjoy the time I spend with the people who are important to me |
| When somethings on my mind, just talking with the people I know can make me feel better |
| When I need someone to help me out, I can usually find someone |

**Supplementary Figure 1.** Ten items used to measure social interactions and support in the HILDA survey


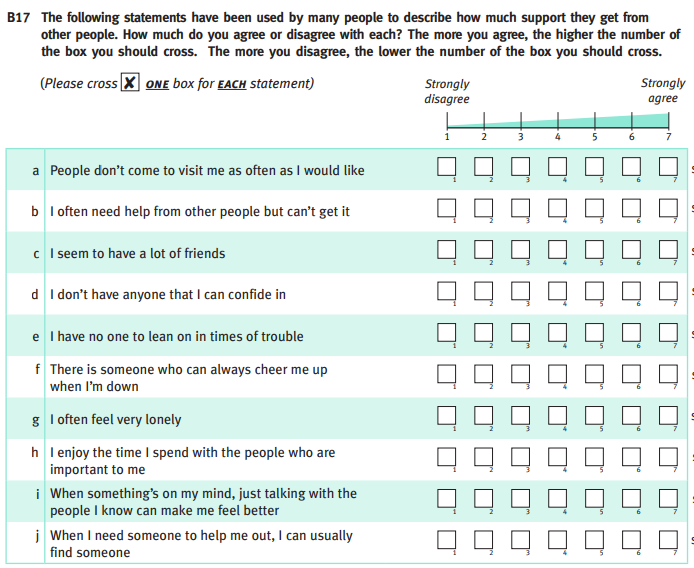


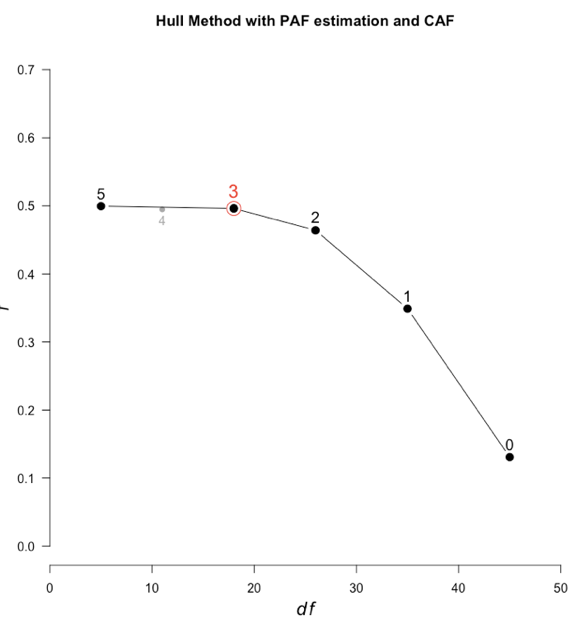
**Supplementary Figure 2a.** Results of the Hull Method in wave 17 (calibration sample)

**Supplementary Figure 2b.** Results of the Hull Method in wave 19 (calibration sample)


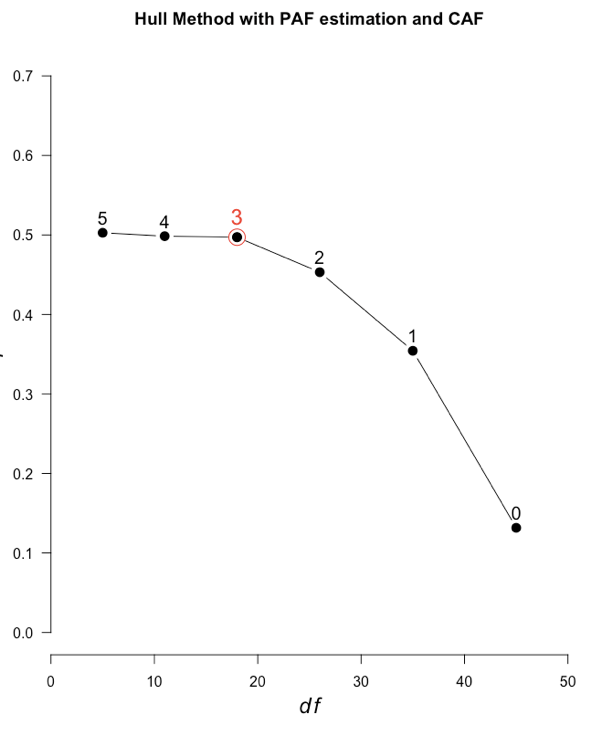

Supplement: Supplementary file 1 — Additional file 1: Table S1. Items included in the loneliness and social isolation sub-scales. Figure 1. Ten items used to measure social interactions and support in the HILDA survey. Figure 2a. Results of the Hull Method in wave 17 (calibration sample). Figure 2b. Results of the Hull Method in wave 19 (calibration sample) [file 12955_2022_1946_MOESM1_ESM.docx]
